# Age of Information Minimization in Multicarrier-Based Wireless Powered Sensor Networks

**DOI:** 10.3390/e27060603

**Published:** 2025-06-05

**Authors:** Juan Sun, Jingjie Xia, Shubin Zhang, Xinjie Yu

**Affiliations:** 1 School of Computer and Data Engineering, NingboTech University, Ningbo 315100, China; sunjuanningbo@163.com (J.S.); shinoyarin@163.com (J.X.); 2 School of Computer Science and Technology, Zhejiang University of Technology, Hangzhou 310023, China; zhangshubin@zjut.edu.cn

**Keywords:** age of information, wireless energy transfer, lyapunov optimization, deep reinforcement learning

## Abstract

This study investigates the challenge of ensuring timely information delivery in wireless powered sensor networks (WPSNs), where multiple sensors forward status-update packets to a base station (BS). Time is partitioned to multiple time blocks, with each time block dedicated to either data packet transmission or energy transfer. Our objective is to minimize the long-term average weighted sum of the Age of Information (WAoI) for physical processes monitored by sensors. We formulate this optimization problem as a multi-stage stochastic optimization program. To tackle this intricate problem, we propose a novel approach that leverages Lyapunov optimization to transform the complex original problem into a sequence of per-time-bock deterministic problems. These deterministic problems are then solved using model-free deep reinforcement learning (DRL). Simulation results demonstrate that our proposed algorithm achieves significantly lower WAoI compared to the DQN, AoI-based greedy, and energy-based greedy algorithms. Furthermore, our method effectively mitigates the issue of excessive instantaneous AoI experienced by individual sensors compared to the DQN.

## 1. Introduction

The timeliness of status updates, originating from diverse stochastic processes and collected by source nodes, plays a crucial role in the performance of numerous real-time systems [1,2,3]. Examples of such applications include safety-critical systems, health monitoring, and environmental surveillance. In these time-sensitive contexts, rapid delivery of sampled information to the destination is necessary. Outdated information can result in suboptimal control decisions and potentially severe consequences. Consequently, the Age of Information (AoI) metric has emerged as a valuable tool for quantifying the freshness and timeliness of status-update data [4]. It represents the elapsed time since the most recent update was generated at the source and successfully received at the destination. The AoI at time *t* is given by t−u(t), where u(t) is the timestamp of the latest received update [5].

Energy limitations in wireless devices (WDs) pose a significant challenge to timely data delivery due to the increased likelihood of packet loss. Wireless energy transfer (WET) has emerged as a key technology to address this issue, allowing for over-the-air recharging of WD batteries and obviating the need for manual replacement [6]. In this work, we focus on the long-term average weighted sum of AoI (WAoI) performance in wireless powered sensor networks (WPSNs), where sensors rely on radio frequency (RF) energy harvested from the base station (BS) to sustain their sensing and communication activities. In WPSNs, the wireless link quality between sensors and the BS is time-varying. Furthermore, the residual energy of sensors and their monitored AoI values vary over time. Consequently, the design of a scheduling policy that minimizes the WAoI at the BS becomes crucial. The network should dynamically select sensors for packet transmission based on a combination of channel quality, energy availability, and AoI. Intuitively, sensors with strong channels, sufficient energy, and high AoI should be prioritized. Additionally, the BS should adapt to network conditions by conducting WET when the communication links are degraded or sensors’ energy is depleted.

### 1.1. Related Work

The introduction of AoI in [4] has spurred extensive research into the characterization of both average and peak AoI [7,8,9,10,11,12,13]. In another line of inquiry, researchers are developing the optimal transmission policy for AoI minimization in communication systems [14,15,16,17,18,19,20,21,22,23,24,25], e.g., broadcast networks [14,15,16], information-update systems employing multiple servers [17], relay-based multi-hop communication systems [18], Internet of Things (Iot) networks [19], UAV-enabled networks [20,21], cognitive radio communication systems [22], multicast networks [23,24], mission-critical vehicular networks [25], and multi-state time-varying networks [26]. Particularly, [14,15,16,17,18,19,20,21,22,23,25] do not consider source nodes utilizing an energy harvesting technique to maintain self-supplying communication operations.

Different from [14,15,16,17,18,19,20,21,22,23,25], another research direction focuses on communication systems that use an energy harvesting technique to power the source nodes. This research aims to determine age-oriented optimal policies for transmitting status-update packets, considering the source nodes’ energy causality constraint. In [27], the authors demonstrate that an energy-dependent threshold policy is optimal for minimizing AoI by triggering new samples. Multiple RF energy harvesting sensors are considered in [28]. To minimize the weighted sum of AoI, the authors employed a DRL framework that concurrently optimize WET and update packet transmissions. In [29], the authors studied relay-based networks, where a source sends status updates to a destination via a relay. Considering the spectrum scarcity, in [30], the authors address the problem of minimizing AoI in cognitive radio networks (CRNs). They derive optimal scheduling actions for both imperfect and perfect spectrum sensing scenarios. Different from traditional fixed energy sources, the work [31] dispatched a UAV to transfer energy to ground sensor nodes. To minimize the average AoI, the authors concurrently optimized energy harvesting durations, the UAV’s trajectory, and data collection time for sensors.

However, none of these prior studies has employed a DRL-based algorithm for the efficient design of freshness-aware WPSNs utilizing multiple subcarriers. In contrast to these works, this paper investigates a scenario where multiple RF-powered source nodes are deployed to sense potentially distinct physical processes and transmit status-update packets across multiple orthogonal subcarriers. For this setting, we provide a novel reinforcement. Before detailing our contributions, we emphasize the key distinctions between our work and those presented in [24,28].

1. Ref. [28] investigates a single-carrier system in contrast to this work, where we focus on a multicarrier system.
2. Ref. [24] considers source nodes with embedded power supplies, whereas our work adopts WPT technology to energize these nodes.
3. In contrast to the approach presented in [24], which aims to minimize the average total transmit power subject to per sensor AoI constraints, our work focuses on minimizing the long-term WAoI.
4. In terms of optimization strategies, ref. [24] relies on conventional numerical methods. In contrast, our work pioneers a scheduling algorithm based on DRL. Moreover, while [28] employs the classical Deep Q-Network (DQN) algorithm, our research introduces a distinctly different DRL algorithm tailored to the specific challenges of our problem.

### 1.2. Contributions

This paper investigates WPSNs for status updates, comprising multiple sensors and a BS, where the BS receives timely information regarding different physical processes monitored by the sensors. The sensors harvest energy from RF signals transmitted by the BS and communicate using orthogonal subcarriers within each time block. The primary contributions of this work are summarized as follows:

1. We formulate the problem of jointly optimizing subcarrier assignment, WET duration, and sensor sampling schedules to minimize the WAoI for diverse physical processes at the BS within a time-sensitive communication system. This is modeled as a multi-stage stochastic optimization problem, subject to energy causality constraints at the sensors.
2. To address this optimization problem, we propose a novel dynamic control algorithm that integrates DRL and Lyapunov optimization techniques. Specifically, Lyapunov optimization is employed to decompose the multi-stage stochastic problem into a sequence of deterministic optimization problems, one for each time block. Subsequently, a DRL algorithm is utilized to determine the optimal scheduling decisions for each time block, with action exploration facilitated by a randomization policy.
3. Extensive simulation results demonstrate the significant performance gains of our proposed algorithm in reducing the WAoI compared to benchmark algorithms, including the DQN, energy-based greedy, and AoI-based greedy schemes. Notably, our DRL algorithm exhibits good convergence performance and eliminates the need for a predefined upper limit for AoI values, unlike the DQN approach.

## 2. System Model and Problem Formulation

### 2.1. Network Model

**Figure 1:**
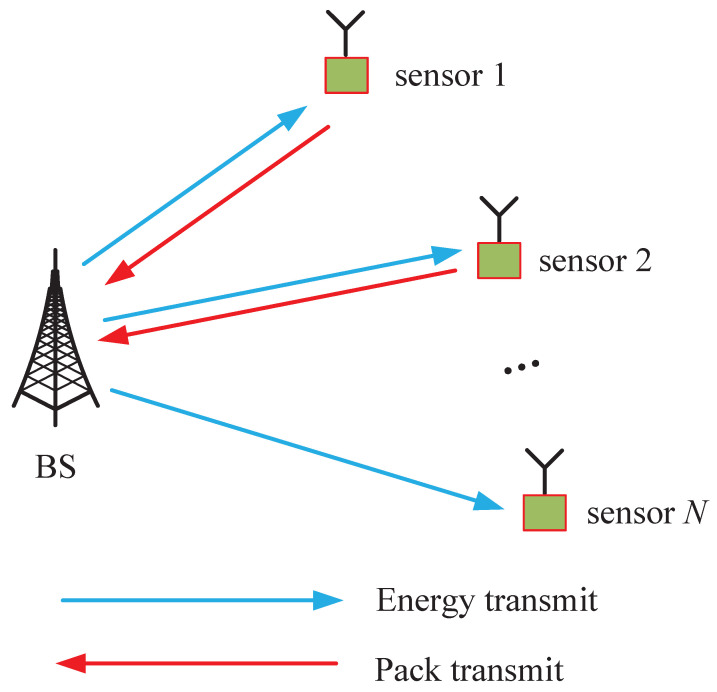
System model.

**Figure 2:**
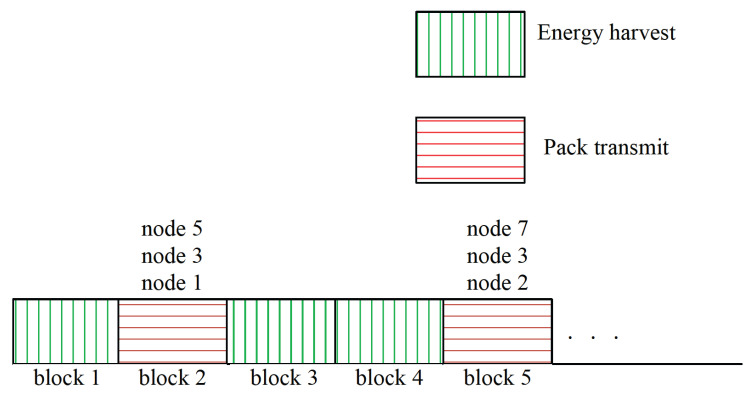
Illustration of the scheduling process for energy transfer and packet transmission in WPSNs.

A real-time monitoring system is considered, as shown in Figure 1, where a BS collects time-critical information from *N* sensors. The sensors are responsible for providing the BS with fresh information about their respective measured processes. Additionally, the sensors share *K* subcarriers, each with a bandwidth of *W* Hz. The BS is assumed to have a stable power supply, whereas each sensor *n* is powered by the RF energy transmitted by the BS in the downlink. This harvested energy is stored in a battery with a finite capacity of $B_{\max,n}$ joules. The communication is divided into discrete time intervals, indexed by t=0,1,2,⋯,T−1. In each time block, either energy transfer or pack transmission is conducted. Figure 2 illustrates a representative schedule, where the BS broadcasts RF energy in time block 0, 2, and 3, and sensors 1, 3, and 5 transmit update packs in time block 1.

We consider a quasi-static fading channel model, where the channel power gain is constant within a time block but varies independently across different time blocks. Let $g_{n,k}\left(t\right)$ and $h_{n,k}\left(t\right)$ represent the channel power gains on subcarrier *k* of the uplink and downlink channels between the BS and sensor *n*, respectively. Additionally, let $A_{n}\left(t\right)$ and $B_{n}\left(t\right)$ represent the AoI of sensor *n*’s monitoring process and its remaining energy, respectively. It should be noted that this paper does not impose an upper bound on the AoI.

### 2.2. State and Action Spaces

At time block *t*, sensor *n*’s state, $s_{n}\left(t\right)$, is defined by its downlink and uplink channel power gains on subcarrier *k*, the AoI of its measured process at the BS, and its battery level, i.e., $s_{n}\left(t\right)≜\left(h_{n,k}\left(t\right),\;g_{n,k}\left(t\right),\;A_{n}\left(t\right),\;B_{n}\left(t\right)\right)\in S_{n}^{a}$. $S_{n}^{a}$ denotes the state space encompassing all combinations of $h_{n,k}\left(t\right)$, $g_{n,k}\left(t\right)$, $A_{n}\left(t\right)$, and $B_{n}\left(t\right)$. Consequently, $s\left(t\right)={\left\{s_{n}\left(t\right)\right\}}_{n\in N}\in S^{a}$ denotes the state of the system at time slot *t*, where $S^{a}$ denotes the state space of the system. Then, at time block *t*, the possible action is expressed as $a\left(t\right)\in A≜\left\{H,\;\underset{K}{\underset{︸}{\left(T_{1},\;T_{2},\;\cdots \right)}},\;\underset{K}{\underset{︸}{\left(T_{1},\;T_{3},\;\cdots \right)}},\;\cdots ,\;\underset{K}{\underset{︸}{\left(T_{i},\;T_{j},\;\cdots \right)}}\right\}$. If a(t)=H, the BS transmits RF energy to the sensors via the downlink. For sensor *n*, the captured energy is expressed as

(1) $$E_{n}^{H}\left(t\right)=\eta Ph_{n,k}\left(t\right),$$

where η denotes the efficiency of the energy harvesting and *P* represents the power transmitted by the BS.

If $a\left(t\right)=\underset{K}{\underset{︸}{\left(T_{i},\;T_{j},\;\cdots \right)}}$, *K* sensor nodes (i,e., node *i*, node *j*, …) send the status-update packs to the BS through the uplink over the *K* subcarriers. The sensors employ a generate-at-will strategy, where data packets are generated immediately following a scheduling decision [32]. It should be noted that when sensor *n* transmits a data packet of size $S_{n}$ to the BS at time block *t*, the energy consumption is denoted by $E_{n}^{C}\left(t\right)$. According to Shannon’s formula, $E_{n}^{C}\left(t\right)$ is expressed as

(2) $$E_{n}^{C}\left(t\right)=\frac{\sigma^{2}}{g_{n,k}\left(t\right)}\left(2^{\frac{S_{n}}{W}}-1\right),$$

where $\sigma^{2}$ represents the noise variance. Therefore, sensor *n* is eligible for data transmission only if its remaining energy satisfies

(3) $$B_{n}\left(t\right)\geq E_{n}^{C}\left(t\right)=\frac{\sigma^{2}}{g_{n,k}\left(t\right)}\left(2^{\frac{S_{n}}{W}}-1\right).$$

At time block *t*, the AoI for different physical processes and the battery level of each sensor are updated after executing decisions as shown in Figure 1. Specifically, when a(t)=H, process *n*’s AoI observed by sensor *n* increments by one and the remaining battery energy of sensor *n* increases by $E_{n}^{H}\left(t\right)$. When $a\left(t\right)=\underset{K}{\underset{︸}{\left(T_{i},\;T_{j},\;\cdots \right)}}$, i.e., the packet is transmitted, and the battery level of *K* sensors scheduled decreases by $E_{n}^{C}\left(t\right)$, while the battery level of other sensors remain unchanged. Meanwhile, the AOI of the physical processes monitored by the selected *K* sensors are set to 1, while the other sensors remain unchanged. Therefore, the dynamic changes in sensor *n*’s remaining energy and the AoI are given, respectively, by

(4) $$B_{n}\left(t+1\right)\;\;=\;\;\left\{\begin{array}{c} B_{n}\left(t\right)\;-\;E_{n}^{C}\left(t\right),\;\;\;\;\\ \mathrm{if}\;\;a\left(t\right)=\underset{K}{\underset{︸}{\left(T_{i},\;T_{j},\;\cdots \right)}}\mathrm{and}\;\;T_{n}\in \underset{K}{\underset{︸}{\left(T_{i},\;T_{j},\;\cdots \right)}},\\ \min\{B_{n}\left(t\right)\;+\;E_{n}^{H}\left(t\right),\;B_{\max,n}\},\;\;\;\;\mathrm{if}\;\;a\left(t\right)=H,\\ B_{n}\left(t\right),\;\;\;\;\;\;\;\;\;\;\mathrm{otherwise}. \end{array}\right.$$

(5) $$A_{n}\left(t+1\right)\;\;=\;\;\left\{\begin{array}{c} 1,\;\;\\ \mathrm{if}\;\;a\left(t\right)=\underset{K}{\underset{︸}{\left(T_{i},\;T_{j},\;\cdots \right)}}\mathrm{and}\;\;T_{n}\in \underset{K}{\underset{︸}{\left(T_{i},\;T_{j},\;\cdots \right)}},\\ A_{n}\left(t+1\right)+1,\;\;\;\;\;\;\mathrm{otherwise}. \end{array}\right.$$

**Figure 3:**
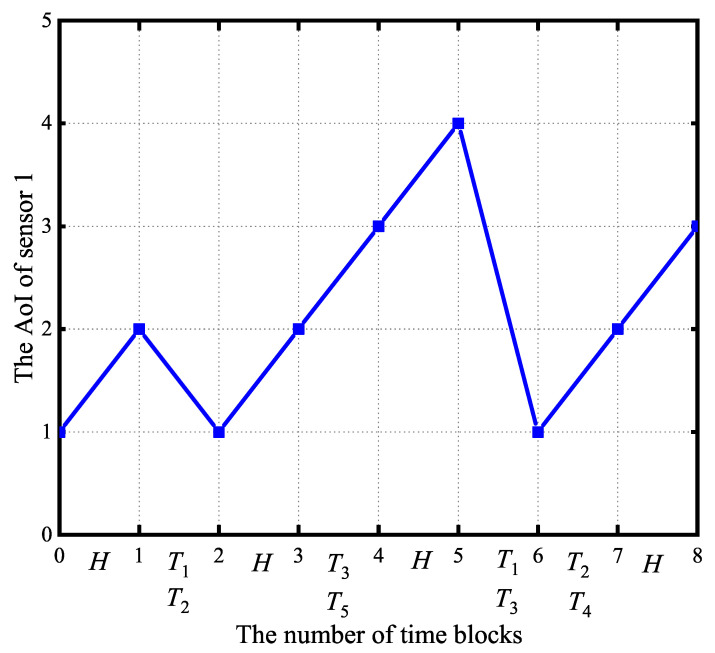
Illustration of sensor’s AOI evolution.

To help visualize (5), Figure 3 shows the AoI evolution of process 1 when taking action over time, where N=5 and K=2. We can observe from Figure 3 that $a\left(1\right)=\left(T_{1},T_{2}\right)$ then $A_{1}\left(2\right)=1$, and $a\left(5\right)=\left(T_{1},T_{3}\right)$ then $A_{1}\left(6\right)=1$. Specifically, the AoI of process 1 is reset to 1 at the start of time blocks 2 and 6, corresponding to status updates transmitted at time blocks 1 and 5. During time blocks 0, 2, 3, 4, 6, and 7, sensor 1 remains inactive (either harvesting energy or idle), causing the AoI of its monitoring process increment by 1 in each of these time blocks.

### 2.3. Problem Formulation

We aim to minimize the WAoI at the BS by finding the optimal policy for action selection at each time block. The policy consists of a set of decision rules {π(0),π(1),…} such that for any time block *t*, π(t) assigns an action a(t)∈A to each possible system state $s\left(t\right)\in S^{a}$. Given policy π and initial state s(0), process *n*’s long-term AoI is given by

(6) $${\bar{A}}_{n}^{\pi }≜\lim\;\sup_{t\to \infty }\frac{1}{T+1}E\left[\sum_{t=0}^{T}A_{n}\left(t\right)|s\left(0\right)\right].$$

Consequently, the WAoI minimization problem for WPSNs is formulated as

(7) $$\begin{array}{cc} (P1):\;\;\; & \pi^{*}=\arg\;\;\min_{\pi }\sum_{n=1}^{N}\beta_{n}{\bar{A}}_{n}^{\pi },\\ \; & s.t.\;\;(3)-(5), \end{array}$$

where $\pi^{*}$ denotes the optimal policy, and the weight $\beta_{n}$ is non-negative and satisfies $\sum_{n=1}^{N}\beta_{n}=1$. Clearly, if π(t)=H, the optimal policy is to allocate all sensors harvesting RF energy from the BS. If $\pi \left(t\right)=\underset{K}{\underset{︸}{\left(T_{i},\;T_{j},\;\cdots \right)}}$, the optimal policy is to allocate *K* sensors send status-update packs to the BS based on each sensor’s battery level, channel state information, and each process *n*’s AoI state monitored by sensor *n*.

Classic optimization techniques like combinatorial optimization and heuristics are not appropriate for this problem due to its long-term and stochastic nature. Primarily designed for deterministic scenarios, these methods can only optimize for the immediate time block, thus struggling to achieve optimal performance over extended periods.

## 3. The Decoupling Strategy for Multi-Stage Stochastic Optimization Based on Lyapunov Theory

This section introduces LODR, an algorithm that combines Lyapunov optimization and DRL, to tackle the problem (P1). We begin by employing Lyapunov optimization to transform the original problem into a series of deterministic problems, one for each time block. For each sensor, we define *N* virtual energy queues, denoted by ${\left\{Q_{n}\left(t\right)\right\}}_{n=1}^{N}$. These queues are initialized with $Q_{n}\left(1\right)=0$ and updated according to the following equation

(8) $$Q_{n}\left(t+1\right)=\max\{Q_{n}\left(t\right)-\nu E_{n}^{C}\left(t\right)+\nu E_{n}^{H}\left(t\right),0\},\forall n,$$

where ν is a scaling coefficient. $Q_{n}\left(t\right)$ functions as a queue, which is incremented by energy harvests $\nu E_{n}^{H}\left(t\right)$ and decremented by energy consumption $\nu E_{n}^{C}\left(t\right)$.

We introduce a queue backlog vector $Q\left(t\right)={\left\{Q_{n}\left(t\right)\right\}}_{n=1}^{N}$ to characterize the energy queue status of all sensors, where $Q_{n}\left(t\right)$ denotes the backlog of the energy queue for sensor *n* at time block *t*. We then define a Lyapunov function L(Q(t)) and its associated drift △L(Q(t)) as [33]

(9) $$L\left(Q\left(t\right)\right)=0.5\sum_{n=1}^{N}Q_{n}{\left(t\right)}^{2},$$

(10) △L(Q(t))=E[L(Q(t+1))−L(Q(t))|Q(t))].

Then, we utilize the Lyapunov-plus-penalty minimization method from [34] to minimize the WAoI subject to the stability constraint of the queue Q(t). Our approach involves minimizing an upper bound on the following drift-plus-penalty expression for time block *t*.

(11) $$\wedge \left(Q\left(t\right)\right)≜△L\left(Q\left(t\right)\right)+V\cdot E\left[\sum_{n=1}^{N}\beta_{n}{\bar{A}}_{n}^{\pi }|Q\left(t\right)\right].$$

The parameter V>0 serves as a control variable, balancing the importance of the penalty term (i.e., the objective function) and the queue backlog sizes. Adjusting V>0 can achieve a desired balance between the objective function value and the sizes of the queue backlogs.

We next establish an upper bound for ∧(Q(t)). To start, we obtain

(12) $$\begin{array}{cc} Q_{n}{\left(t+1\right)}^{2}= & Q_{n}{\left(t\right)}^{2}+2Q_{n}\left(t\right)\left(E_{n}^{H}\left(t\right)-E_{n}^{C}\left(t\right)\right)\\ \; & +{\left(E_{n}^{H}\left(t\right)-E_{n}^{C}\left(t\right)\right)}^{2}. \end{array}$$

Performing a summation over all queues, we obtain

(13) $$\begin{array}{cc} \; & 0.5\sum_{n=1}^{N}Q_{n}{\left(t+1\right)}^{2}-0.5\sum_{n=1}^{N}Q_{n}{\left(t\right)}^{2}\\ \; & =0.5\sum_{n=1}^{N}{\left(E_{n}^{H}\left(t\right)-E_{n}^{C}\left(t\right)\right)}^{2}+\sum_{n=1}^{N}Q_{n}\left(t\right)\left(E_{n}^{H}\left(t\right)-E_{n}^{C}\left(t\right)\right). \end{array}$$

Applying the conditional expectation operator to both sides of (13) [35], we obtain

(14) $$\begin{array}{c} △L\left(Q\left(t\right)\right)\leq B+\sum_{n=1}^{N}Q_{n}\left(t\right)E\left[\left(E_{n}^{H}\left(t\right)-E_{n}^{C}\left(t\right)\right)|Q\left(t\right)\right]. \end{array}$$

Here, *B* is a constant given as

(15) $$\begin{array}{cc} \; & 0.5\sum_{n=1}^{N}E\left[({\left(E_{n}^{H}\left(t\right)-E_{n}^{C}\left(t\right)\right)}^{2}\right]\\ \; & \leq 0.5\sum_{n=1}^{N}\left[{\left(E_{n,\max}^{H}\left(t\right)\right)}^{2}+{\left(E_{n,\max}^{C}\left(t\right)\right)}^{2}\right]≜B, \end{array}$$

where $E_{n,\max}^{H}\left(t\right)$ is defined as sensor *n* harvesting RF energy at time block *t*, and $E_{n,\max}^{C}\left(t\right)$ is defined as sensor *n* forwarding data at time block *t*. Therefore, the drift-plus-penalty expression in (11) is upper-bounded by

(16) $$\begin{array}{cc} \; & B+\sum_{n=1}^{N}Q_{n}\left(t\right)E\left[\left(E_{n}^{H}\left(t\right)-E_{n}^{C}\left(t\right)\right)|Q\left(t\right)\right]\\ \; & +V\cdot E[\sum_{n=1}^{N}\beta_{n}{\bar{A}}_{n}^{\pi }|Q\left(t\right)]. \end{array}$$

Applying the principle of opportunistic expectation minimization [36] at time block *t*, the scheduling decision is made based on the observed queue backlog Q(t) to minimize the upper bound established in (16). Noting that the control action decision at time block *t* is only affected by the second and third terms, the algorithm’s action selection at time block *t* is predicated on the minimization of the subsequent expression, derived after removing constants from the initial observation:

(17) $$\begin{array}{cc} V\sum_{n=1}^{N}\beta_{n}{\bar{A}}_{n}^{\pi } & +\sum_{n=1}^{N}Q_{n}\left(t\right)E_{n}^{H}\left(t\right)-\sum_{n=1}^{N}Q_{n}\left(t\right)E_{n}^{C}\left(t\right), \end{array}$$

where the second term corresponds to RF energy harvesting by sensor *n*, and the third term indicates that sensor *n* is selected for transmission. Then, the original problem (P1) is reformulated as

(18) $$\begin{array}{cc} (P2):\;\min\;\Delta (s(t),\pi (t))= & V\sum_{n=1}^{N}\beta_{n}{\bar{A}}_{n}^{\pi }\\ \; & +\sum_{n=1}^{N}Q_{n}\left(t\right)E_{n}^{H}\left(t\right)\\ \; & -\sum_{n=1}^{N}Q_{n}\left(t\right)E_{n}^{C}\left(t\right),\\ s.t.\;\;\;(3)-(5). \end{array}$$

If problem (7) admits a feasible solution, relying solely on channel-only policies is sufficient to approach the optimal performance with arbitrary precision. Given the assumption of stationary and independent and identically distributed (i.i.d.) channels across time slots, the feasibility of problem (7) implies that for any v>0, a policy relying solely on channel state information exists that satisfies

(19) $$\sum_{n=1}^{N}E\left(\beta_{n}{\bar{A}}_{n}^{\pi }\right)\leq G^{\mathrm{opt}}+v,$$

where $G^{\mathrm{opt}}$ denotes the optimal value of WAoI.

**Proof.**  See proof of Theorem 4.5 in [Ref. [33], Appendix 4.A].    □

Subsequently, the primary obstacle lies in the effective solution of problem (P2) for each time block to achieve the WAoI minimization. For the solution of (P2), we consider the system state $s\left(t\right)≜{\left\{h_{n,k}\left(t\right),g_{n,k}\left(t\right),A_{n}\left(t\right),B_{n}\left(t\right)\right\}}_{n=1}^{N}$ at time block *t*, encompassing downlink and uplink channel gains, the AoI of sensor *n*’s monitoring process, and the remaining energy of sensor *n*. The scheduling control action is then determined based on this state. In general, obtaining the optimal policy requires enumerating $C_{N}^{K}+1$ scheduling actions, a computationally intensive task for moderate *N* and *K* values. Alternative search-based techniques, including block coordinate descent and branch-and-bound, also suffer from high computational cost. To address the challenge of online decision-making in dynamically varying channel environments, we introduce LODR, a based-DRL algorithm. In the following, we describe the LODR algorithm to solve (P2) efficiently.

## 4. Lyapunov-Guided DRL for Online Scheduling Decisions

**Figure 4:**
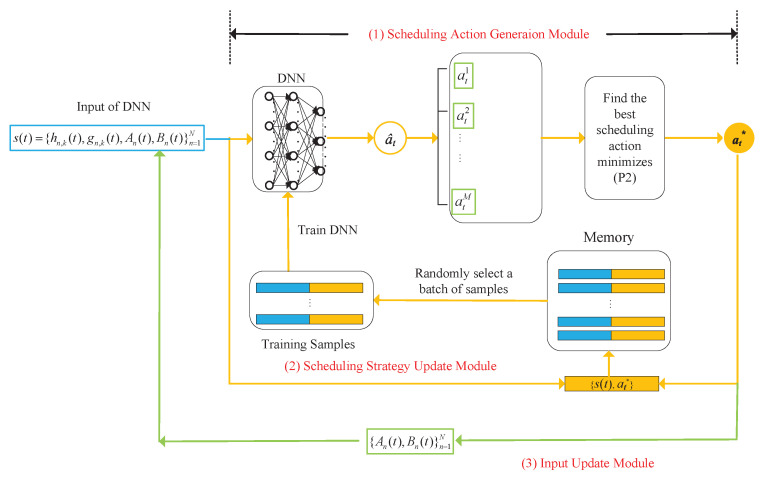
The structure of the proposed LODR algorithm.

Figure 4 shows the architectural framework of the LODR algorithm. It consists of three core modules: the scheduling action generation module, scheduling policy update module, and input update module. The scheduling action generation module begins by receiving the current system state, denoted as s(t). Subsequently, it generates a set of potential scheduling actions. From this set, an evaluation process is executed, resulting in the selection of $a_{t}^{*}$, the action deemed most advantageous. The policy of the scheduling action generation mechanism is refined over time by the scheduling policy update module. Following the execution of the scheduled actions, the input update module changes the state of battery remaining energy of sensors and their monitoring processes’ AoI. A sequential iterative process is followed by these three modules through successive interactions with the stochastic environment ${\left\{h_{n,k}\left(t\right),g_{n,k}\right\}}_{n=1}^{N}$, as outlined in the subsequent sections.

(1) Scheduling Action Generation Module: The DNN, parameterized by $\theta_{t}$, is employed for scheduling action synthesis. When t=1, the parameters $\theta_{t}$ are initialized stochastically according to a Gaussian distribution with zero mean. Subsequent to the DNN’s production of a real-valued scheduling output ${\hat{a}}_{t}$, a discretization process is performed to formulate a set of binary-valued scheduling operations. Specifically, we set *K* largest values of ${\hat{a}}_{t}$ to 1 and the rest to 0, where 1 indicates that the corresponding sensor is selected to send the status update to the BS, while 0 represents the sensor remaining idle. Furthermore, we generate another *M* random actions and one fixed action {0,0,⋯,0}, together with the above DNN output action as candidate actions. Here, {0,0,⋯,0} represents all the sensors harvesting RF energy from the BS.

It is established by the universal approximation theorem that a neural network with a single hidden layer of adequate size is capable of approximating any continuous function *f*, provided that an appropriate activation function is employed, such as the ReLU, tanh, or sigmoid [37] functions. In this implementation, the ReLU activation function is employed within the hidden layers, where the relationship between the neuron’s output *y* and input *x* is defined by y=max{v,0}. For the output layer, the sigmoid activation function is implemented, resulting in the relaxed scheduling action ${\hat{a}}_{t}$ being constrained to the interval (0,1).

It is noted that each potential action yields Δ(s(t),π(t)), which represents the performance metric, through the solution of problem (P2), a defined optimization problem. Consequently, the optimal scheduling action $a_{t}^{*}$ at time block *t* is determined as

(20) $$a_{t}^{*}=\arg\;\;\;\min\Delta \left(s\left(t\right),\pi \left(t\right)\right).$$

(2) Scheduling Policy Update Module: The scheduling solution obtained in (19) will be used to update the scheduling policy of the DNN. To facilitate training, a memory repository of bounded capacity, starting in an empty state, is maintained. In the *t*th time block, a fresh training sample $\left(s\left(t\right),a_{t}^{*}\right)$ is incorporated into the memory buffer. When the memory buffer is at maximum capacity, a first-in-first-out (FIFO) replacement policy is enacted, with the oldest sample being replaced by the newest.

Leveraging the data samples stored within the memory, the training process of the DNN is conducted, an approach recognized as experience replay [38]. The stochastic selection of a training data batch $\left\{\left(s\left(\tau \right),a_{\tau }^{*}\right)|\tau \in \Gamma_{t}\right\}$ is performed from the memory during the *t*th temporal iteration, defined by the set of temporal indices $\Gamma_{t}$. The DNN’s parameter $\theta_{t}$ is updated via the Adam optimization algorithm to minimize the mean cross-entropy loss as

(21) $$\begin{array}{cc} L(\theta_{t}) & =\\ \; & -\frac{1}{|\Gamma_{t}|}\sum_{\tau \in \Gamma_{t}}({\left(a_{\tau }^{*}\right)}^{T}\log f_{\theta_{t}}\left(s\left(\tau \right)\right)\\ \; & +\left(1-a_{\tau }^{*}\right)\log\left(1-f_{\theta_{t}}\left(s\left(t\right)\right)\right)), \end{array}$$

where $|\Gamma_{t}|$ represents the number of elements within $\Gamma_{t}$, ${\left(\cdot \right)}^{T}$ indicates the operation of transpose, and the log function represents the application of the logarithm to each element of a vector. The DNN undergoes periodic retraining every ζ time frames, triggered by the accumulation of a predetermined volume of fresh data.

(3) Input Update Module: Based on the scheduling action generation module, the system executes the scheduling action $a_{t}^{*}$, then the battery level of sensor *n*$B_{n}\left(t+1\right)$ is updated using (4), the AoI $A_{n}\left(t+1\right)$ updates using (5), and the virtual energy queues ${\left\{Q_{n}\left(t+1\right)\right\}}_{n=1}^{N}$ updates using (8). Following the observation of wireless channel gains ${\left\{h_{n,k}\left(t+1\right),g_{n,k}\left(t+1\right)\right\}}_{n=1}^{N}$, the system utilizes the composite input $s\left(t+1\right)=\{h_{n,k}\left(t+1\right),g_{n,k}\left(t+1\right),A_{n}\left(t+1\right),B_{n}\left(t+1\right)\}$ to drive the DNN, thereby triggering a new iterative cycle starting from the scheduling action generation module in Step 1).

In summary, through successive iterations, the DNN refines its scheduling policy by learning from optimal state-action pairs, $\left(s\left(t\right),a_{t}^{*}\right)$, thereby enhancing decision-making over time. Due to the limitation imposed by the finite replay memory, the DNN’s learning is restricted to the most recent data samples, which reflect the latest scheduling policies. Through continuous feedback and adaptation, this closed-loop reinforcement learning framework optimizes the scheduling policy, leading to convergence. Algorithm 1 delineates the methodology employed to determine the optimal scheduling policy for (P1).
**Algorithm 1:** LODR algorithm to solve the AoI minimization problem.
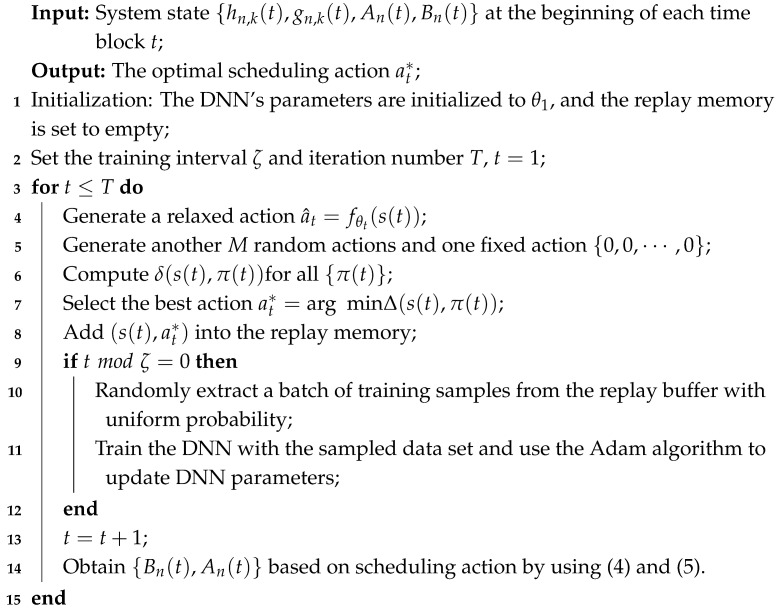


## 5. Performance Evaluation

The proposed LODR algorithm’s performance was evaluated by comparing it with the DQN, energy-based greedy, and AoI-based greedy algorithms. Specifically, for the energy-based and AoI-based greedy algorithms, on the premise of ensuring the minimum energy requirement, we arranged *K* sensors with relatively large battery level and high AoI to send status-update packs to the BS.

### 5.1. Experimental Settings

This subsection details the simulation parameters. The channel model incorporates both small-scale fading and path loss effects. The downlink and uplink channel gains between the BS and sensor *n*, denoted by $h_{n,k}$ and $g_{n,k}$, are modeled as random variables. Specifically, $h_{n,k}$ and $g_{n,k}$ are given by $h_{n,k}=\Upsilon \Psi_{1}^{2}d_{n,k}^{-\kappa }$ and $g_{n,k}=\Upsilon \Psi_{2}^{2}d_{n,k}^{-\kappa }$. Υ is the reference distance (1 m) signal power gain, $d_{n,k}^{-\kappa }$ is the distance between the BS and sensor *n*, and −κ is the path loss exponent. $\Psi_{1}^{2}$ and $\Psi_{2}^{2}$ represent independent, exponentially distributed (mean 1) small-scale fading gains. Unless otherwise stated, the primary simulation parameters are as follows: $d_{1}=20$, $d_{2}=25$, $d_{3}=40$, $d_{4}=15$, $d_{5}=50$, $d_{6}=10$, $d_{7}=45$, $S_{n}=15$, $B_{n,\max}=0.3$ mJ, K=2, and $A_{n}\left(1\right)=2$. $d_{n}$ represents the distance between sensor *n* to the BS.

The DNN employed, whose architecture is specified in Table 1, processed 4N input features. The network’s core consisted of two hidden layers, the first with 120 neurons and the second with 80, both employing the ReLU activation function. The final layer, responsible for generating N+1 outputs, utilized a sigmoid activation. Table 2 shows the simulation parameters for the LODR algorithm.

The state space for the DQN is constructed by discretizing system parameters. Five levels are used to represent both the uplink and downlink channel gains and four levels for the remaining battery energy.

### 5.2. Training Loss for LODR Algorithm

**Figure 5:**
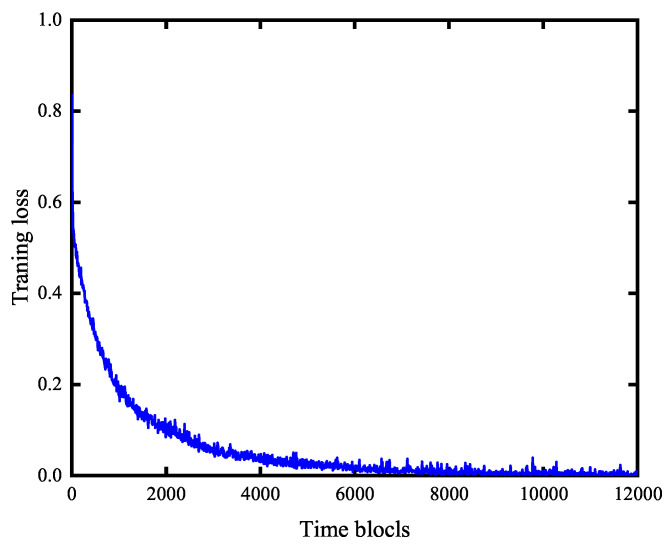
LODR algorithm’s training loss.

Figure 5 presents the training loss of LODR as a function of training steps for a network with N=7 and K=2. The gradual decrease and subsequent convergence of the loss function to a low value demonstrate LODR’s ability to automatically adapt its scheduling policy and reach to the optimal value. The DNN within LODR reaches a stable state within approximately 8000 time blocks, indicating rapid convergence. This convergence behavior is consistently observed in simulations with a larger number of sensors. In contrast, the DQN exhibits significantly slower convergence, or even a lack thereof, as the state space expands. For instance, with seven sensors, discretizing the uplink and downlink channel gains into six and five levels, the battery energy into four levels, and the maximum AoI setting to 5 results in a state space of ${\left(6*5*4*5\right)}^{7}$. Such a vast state space requires extensive exploration for Q-value stabilization, leading to slow convergence. Furthermore, even upon convergence, the DQN algorithm’s loss remains comparatively high.

### 5.3. Impact of M and ζ

**Figure 6:**
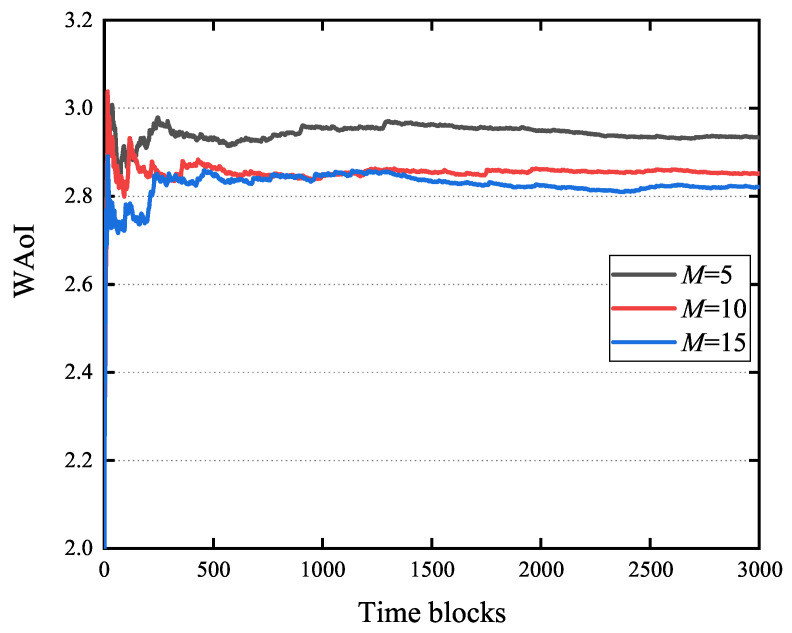
Impact of *M* on WAoI.

Figure 6 illustrates the influence of the number of random actions *M* on the WAoI, employing a parameter configuration consistent with that used in Figure 5. This figure demonstrates rapid convergence of the WAoI for all three values of *M* considered. Furthermore, at 3000 time blocks, the WAoI for M=15 is 3.5% greater than that observed for M=5. This suggests that the number of random actions has a limited impact on the WoI. Meanwhile, as *M* increases, the WAoI decreases.

**Figure 7:**
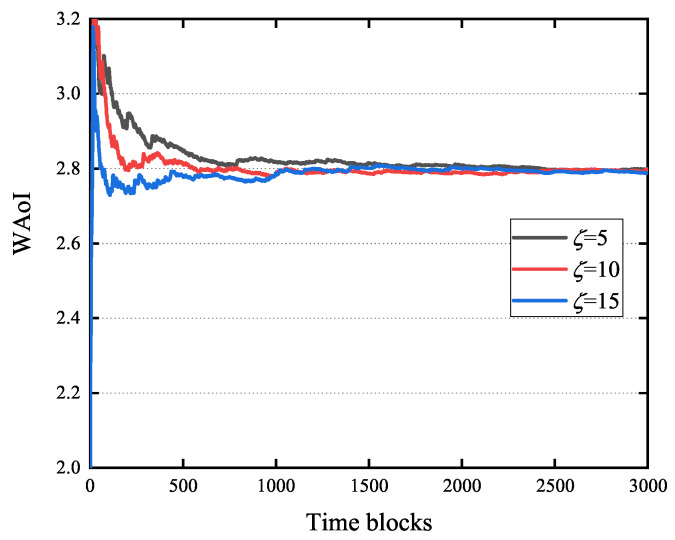
Impact of training interval ζ on the WAoI.

Figure 7 shows the effect of the training interval ζ on the WAoI, using parameters consistent with Figure 5. The WAoI converge to similar values for ζ=10, ζ=20, and ζ=30 after 3000 time slots. ζ=10 was therefore selected for subsequent experiments.

### 5.4. The WAoI of LODR

**Figure 8:**
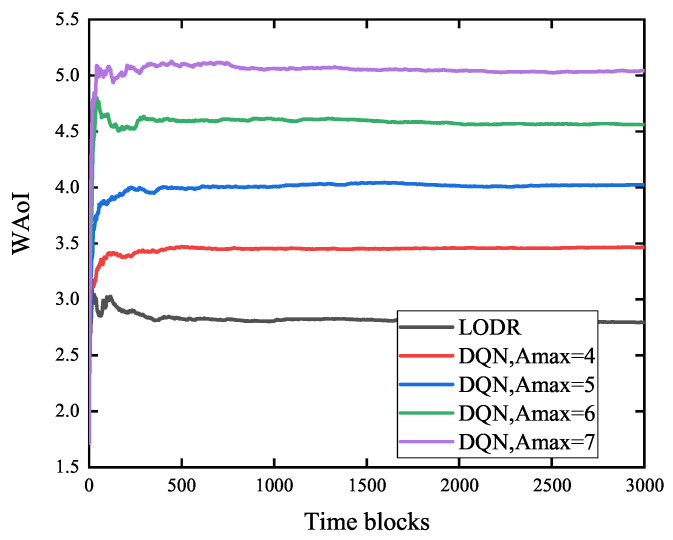
Comparing LODR and DQN in terms of WAoI.

Figure 8 shows the WAoI for LODR and the DQN, where the parameter setup is similar to that in Figure 5. From Figure 8, we can draw two conclusions. First, clearly the WAoI of our proposed LODR algorithm is superior to the classic DQN algorithm. Second, for the DQN, as $A_{\max}$ increases, the system performance deteriorates. This is because the higher the degree of information stale (e.g., $A_{\max}$ is larger) that the system can tolerate, the greater AoI is likely to be. Thus, the setting of $A_{\max}$ obviously affects the performance of the DQN.

**Figure 9:**
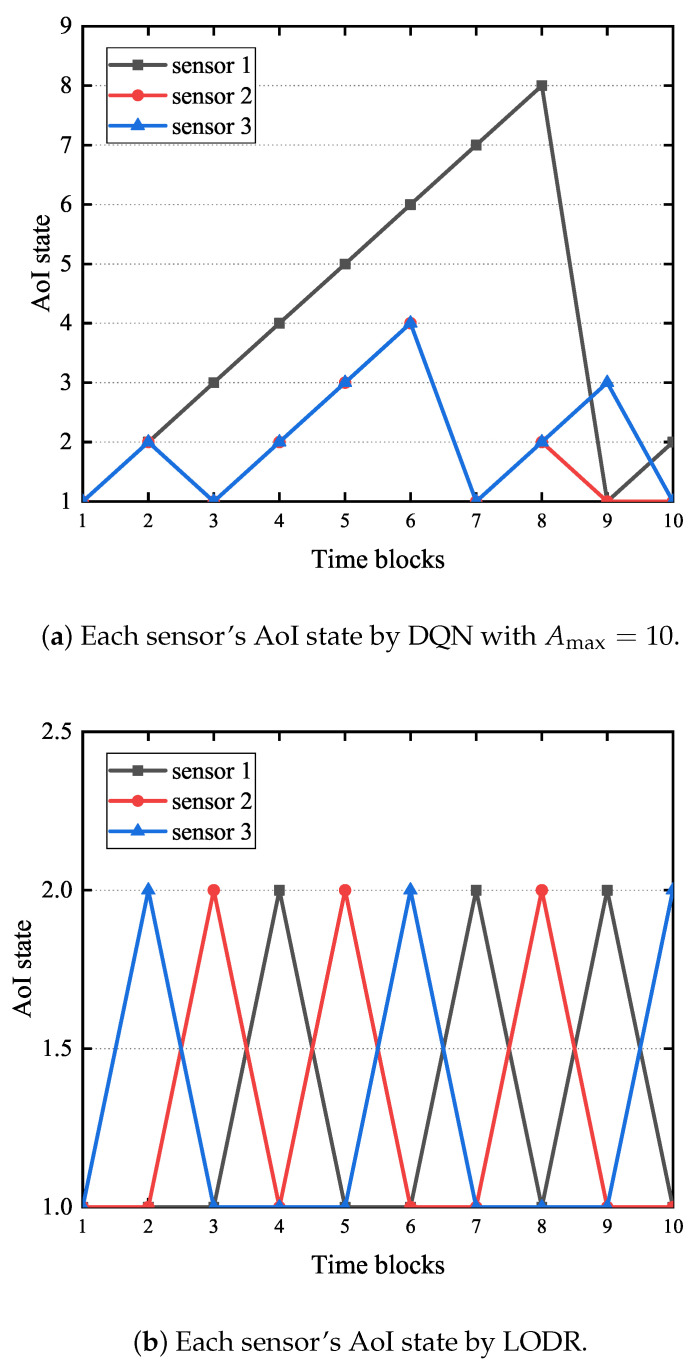
The comparison of each sensor’s AoI state for LODR and the DQN.

Figure 9 illustrates a comparison of AoI evolution for each sensor using LODR and the DQN. As shown in Figure 9, LODR achieves greater AoI stability for all sensors than the DQN. Using the DQN, sensor 1’s AoI reaches 8 by time block 8, and the status-update packet is sent at time block 9. Sensors 2 and 3 exhibit significantly lower peak AoI values, at 50% and 37.5% of sensor 1’s peak, respectively. Notably, the maximum AoI values for sensors 2 and 3 are significantly lower, at only 50% and 37.5% of sensor 1’s peak. This is due to the DQN algorithm’s failure to converge to an optimal policy. Conversely, LODR maintains a maximum AoI of 2 for all sensors, demonstrating its effective dynamic scheduling. Additionally, we observe the consequent time blocks, and the AoI of each sensor is the same in the steady state. It should be noted that this only applies to the current parameter setup. When we change the parameters, each sensor has a different AOI.

**Figure 10:**
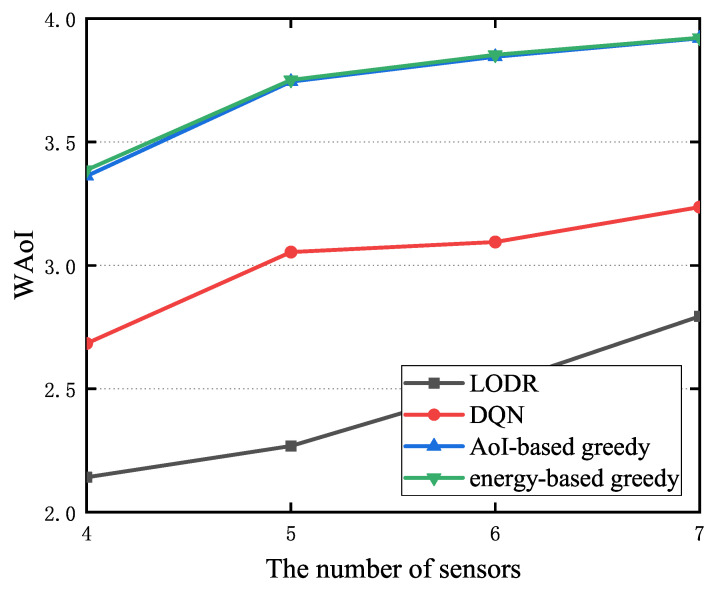
The WAoI for the LODR, DQN, AoI-based, and energy-based algorithms.

Figure 10 shows the WAoI for the LODR, DQN, AoI-based greedy, and energy-based greedy algorithms. As illustrated in Figure 10, LODR outperforms the other three algorithms. Our simulations revealed that the DQN struggles to converge with an increasing number of sensors. When the number of sensors is large but the selection is limited, considering the case of selecting two nodes, the stability of the DQN’s state is compromised. After multiple rounds of selection, there will inevitably be several sensors with continuously increasing AoI until the upper limit is reached. However, now, if two nodes are selected based on a greedy algorithm, the senor with the maximum energy will undoubtedly have the maximum AoI. At this point, the two greedy algorithms degenerate into one, resulting in similar AoI. Additionally, the WAoI increases with the number of sensors. Larger sensor deployments result in reduced transmission probability and increased WAoI.

**Figure 11:**
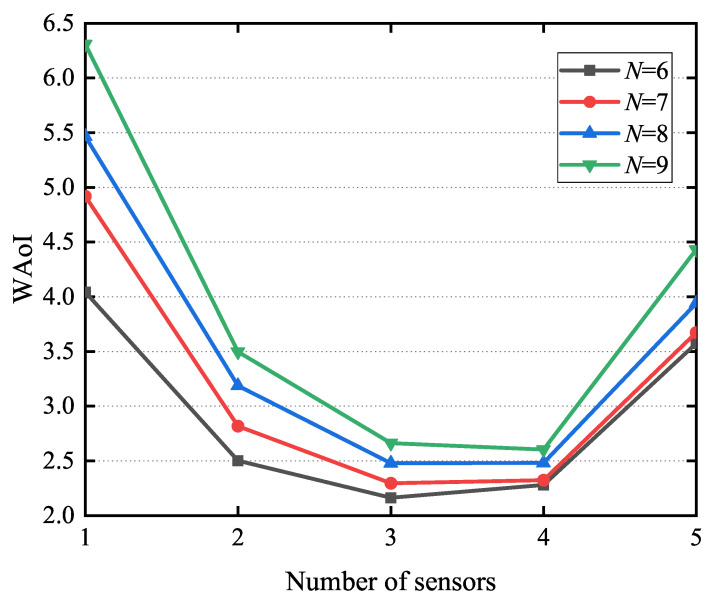
The influence of the number of subcarriers *K* on WAoI.

Figure 11 illustrates how the WAoI varies with the number of subcarriers *k*, where the number of sensors *N* is set to 6, 7, 8, and 9. In Figure 11, we can observe that the WAoI exhibits a trend of initial decrease followed by a subsequent increase. Larger *K* values correlate with a higher chance of repeated sensor selections. When *K* is below about half of *N*, the probability of repeating the same sensor is less likely. The sensors are used evenly, entering a charging state slowly and reducing the WAoI with increasing *K*. However, when *K* surpasses about half of *N*, reusing the same sensor in different time blocks accelerates energy consumption, leading to faster charging and an increase in the WAoI with growing *K*.

**Figure 12:**
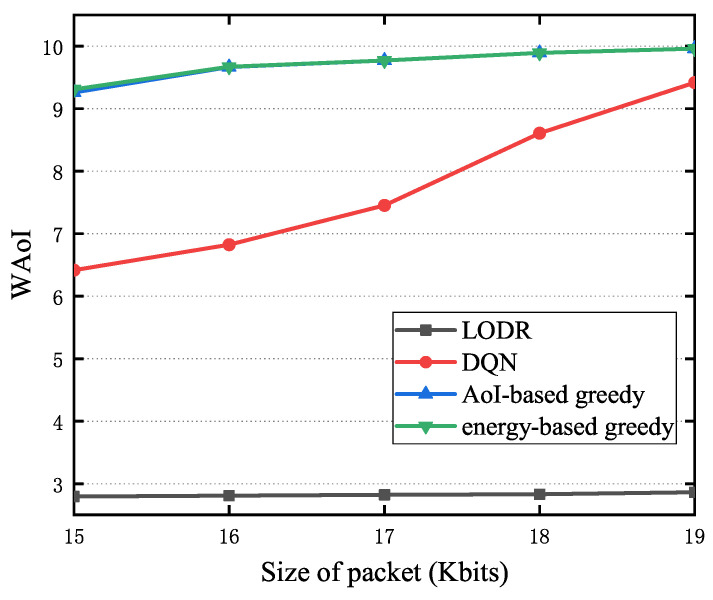
The impact of the status-update packet size on WAoI.

Figure 12 illustrates the influence of the size of the status-update pack on WAoI for the LODR, AoI-based greedy, energy-based greedy, and DQN methods with $A_{\max}=10$. It is observed that the WAoI exhibits a positive correlation with the size of status-update pack. This is because the increased energy demands of larger status-update packets necessitate longer energy harvesting periods, consequently leading to a higher AoI.

**Figure 13:**
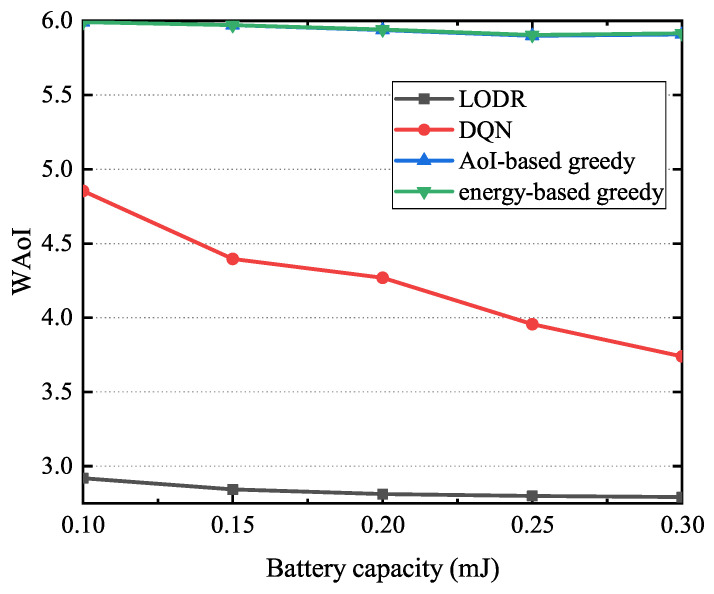
The impact of the capacity of batteries on WAoI.

Figure 13 illustrates the relationship between the battery capacity and WAoI for the LODR, AoI-based greedy, energy-based greedy, and DQN methods with $A_{\max}=6$. From Figure 13, we can see that the WAoI exhibits an inverse relationship with the battery capacity. This is because as the battery capacity increases, so does the energy storage capacity of sensors, which in turn increases the probability of status-update transmissions.

## 6. Conclusions

We considered the WPSNs where multiple sensors send status-update packets to the BS, aiming to minimize the WAoI of different processes at the BS. Specifically, we first formulated the WAoI minimization problem as a multi-stage stochastic optimization problem subject to energy causality constraints. Second, we designed the algorithm LODR, which jointly utilizes DRL and Lyapunov optimization. Compared to the classic DQN algorithm, LODR has better convergence performance, alleviates the problem that some sensors may have large AoIs, and is able to effectively schedule the energy transfer and packet transmit with the dynamic of network state. LODR achieves smaller WAoI than the DQN, AoI-based greedy, and energy-based greedy algorithms. Additionally, the WAoI is an increasing function with respect to the size of status-update packets, and it is a decreasing function with respect to the capacity of batteries.

## Figures and Tables

**Table 1:** The DNN architecture.

Layers	Number of Neurons	Activation Function
Input layer	4N	/
Hidden layer 1	120	ReLU
Hidden layer 2	80	ReLU
HOutput layer	N+1	Sigmoid

**Table 2:** Simulation parameters of LODR.

Simulation Parameter	Values
Learning rate	0.01
Training interval	10
Memory size	1024
Batch size	128

## Data Availability

The data presented in this study are available on request from the corresponding author.

## Abbreviations

The following abbreviations are used in this manuscript:

AoI	Age of Information
WPSNs	Wireless powered sensor networks
WAoI	Long-term average weighted sum of Age of Information
RF	Radio frequency
